# Bis(10-meth­oxy­benzo[*h*]quinolinium) tetra­chloridozinc

**DOI:** 10.1107/S1600536811055462

**Published:** 2012-01-11

**Authors:** Zhenming Dong, Bo Liu

**Affiliations:** a School of Chemistry and Chemical Engineering, Shanxi University, Taiyuan 030006, People’s Republic of China; b Institute of Chemistry, School of Science, Beijing Jiaotong University, Beijing 100044, People’s Republic of China

## Abstract

In the title compound, (C_14_H_12_NO)_2_[ZnCl_4_], the benzo[*h*]quinolinium groups are approximately planar, with maximum deviations of 0.049 (8) and 0.056 (9) Å. The meth­oxy groups are stabilized by intra­molecular N—H⋯O hydrogen bonds. The structure also exhibits weak inter­molecular N—H⋯Cl hydrogen bonds between the cations and anions. π–π inter­actions are present between the pyridinium and benzene rings [centroid–centroid distances = 3.640 (4), 3.728 (5) and 3.628 (5) Å].

## Related literature

For background to quinoline derivatives, see: Kouznetsov *et al.* (2005 ▶). For related complexes, see: Guo *et al.* (2007 ▶).
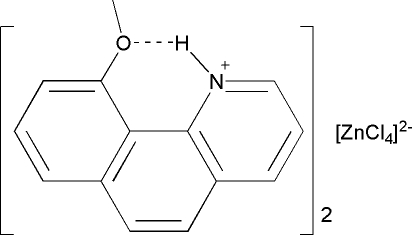


## Experimental

### 

#### Crystal data

(C_14_H_12_NO)_2_[ZnCl_4_]
*M* *_r_* = 627.66
Triclinic, 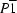

*a* = 8.3846 (15) Å
*b* = 9.6352 (18) Å
*c* = 18.348 (3) Å
α = 91.810 (3)°
β = 92.508 (3)°
γ = 114.967 (3)°
*V* = 1340.4 (4) Å^3^
*Z* = 2
Mo *K*α radiation
μ = 1.35 mm^−1^
*T* = 293 K
0.30 × 0.20 × 0.20 mm

#### Data collection

Bruker APEX CCD diffractometer
Absorption correction: multi-scan (*SADABS*; Sheldrick, 1996 ▶) *T*_min_ = 0.688, *T*_max_ = 0.775
5013 measured reflections
4177 independent reflections
3432 reflections with *I* > 2σ(*I*)
*R*_int_ = 0.028

#### Refinement

*R*[*F* ^2^ > 2σ(*F* ^2^)] = 0.073
*wR*(*F* ^2^) = 0.220
*S* = 1.18
4177 reflections
337 parameters
H-atom parameters constrained
Δρ_max_ = 0.92 e Å^−3^
Δρ_min_ = −0.85 e Å^−3^

### 

Data collection: *SMART* (Bruker, 2007 ▶); cell refinement: *SAINT* (Bruker, 2007 ▶); data reduction: *SAINT*; program(s) used to solve structure: *SHELXS97* (Sheldrick, 2008 ▶); program(s) used to refine structure: *SHELXL97* (Sheldrick, 2008 ▶); molecular graphics: *XP* in *SHELXTL* (Sheldrick, 2008 ▶); software used to prepare material for publication: *SHELXTL*.

## Supplementary Material

Crystal structure: contains datablock(s) I, global. DOI: 10.1107/S1600536811055462/hy2499sup1.cif

Structure factors: contains datablock(s) I. DOI: 10.1107/S1600536811055462/hy2499Isup2.hkl

Additional supplementary materials:  crystallographic information; 3D view; checkCIF report

## Figures and Tables

**Table 1:** Hydrogen-bond geometry (Å, °)

*D*—H⋯*A*	*D*—H	H⋯*A*	*D*⋯*A*	*D*—H⋯*A*
N1—H1⋯O1	0.86	1.95	2.612 (7)	133
N1—H1⋯Cl1^i^	0.86	2.68	3.319 (6)	132
N2—H2⋯O2	0.86	1.93	2.598 (7)	134
N2—H2⋯Cl2^ii^	0.86	2.84	3.472 (6)	132

Symmetry codes: (i) 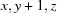; (ii) 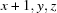.

**Fig. 1.**
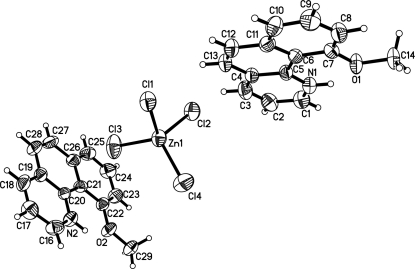
Molecular structure of the title compound. Displacement ellipsoids are drawn at the 50% probability level.

## supplementary crystallographic information

### Comment

Quinoline derivatives represent a major class of heterocycles, and a number of preparations have been known since the late 1800s (Kouznetsov *et al.*, 2005). The quinoline ring system occurs in various natural products, especially in alkaloids (Kouznetsov *et al.*, 2005). In the course of exploring new quinoline complexes (Guo *et al.*, 2007), we obtained the title compound and the synthesis and structure are reported here.

In the title compound (Fig. 1), the benzo[*h*]quinolinium groups are planar, with maximum deviations from the average planes of 0.049 (8) and 0.056 (9) Å, respectively. The methoxy groups are stabilized by intramolecular N—H···O hydrogen bonds (Table 1). The structure also exhibits week intermolecular N—H···Cl hydrogen bonds between the cations and anions. π–π interactions are present between the pyridinium and benzene rings [centroid–centroid distances = 3.640 (4), 3.728 (5) and 3.628 (5) Å].

### Experimental

10-Methoxybenzo[*h*]quinoline (0.30 g, 1.43 mmol) was dissolved in THF (20 ml) and ZnCl_2_ (0.20 g, 1.48 mmol) was added. The mixture was heated with stirring at reflux temperature for 8 h, then cooled to 333 K and filtered. The filtrate was condenced to get yellow crystals suitable for X-ray analysis.

### Refinement

All H atoms were placed in geometrically calculated positions and refined using a riding model, with C—H = 0.93 (aromatic) and 0.96 (methyl) and N—H = 0.86 Å and with *U*_iso_(H) = 1.2(1.5 for methyl)*U*_eq_(C, N).

### Crystal data

**Table:**

(C_14_H_12_NO)_2_[ZnCl_4_]	*Z* = 2
*M**_r_* = 627.66	*F*(000) = 640
Triclinic, *P*1	*D*_x_ = 1.555 Mg m^−^^3^
Hall symbol: -P 1	Mo *K*α radiation, λ = 0.71073 Å
*a* = 8.3846 (15) Å	Cell parameters from 2730 reflections
*b* = 9.6352 (18) Å	θ = 2.2–27.5°
*c* = 18.348 (3) Å	µ = 1.35 mm^−^^1^
α = 91.810 (3)°	*T* = 293 K
β = 92.508 (3)°	Block, yellow
γ = 114.967 (3)°	0.30 × 0.20 × 0.20 mm
*V* = 1340.4 (4) Å^3^	

### Data collection

**Table:**

Bruker APEX CCD diffractometer	4177 independent reflections
Radiation source: fine-focus sealed tube	3432 reflections with *I* > 2σ(*I*)
graphite	*R*_int_ = 0.028
φ and ω scans	θ_max_ = 24.3°, θ_min_ = 2.3°
Absorption correction: multi-scan (*SADABS*; Sheldrick, 1996)	*h* = −9→9
*T*_min_ = 0.688, *T*_max_ = 0.775	*k* = −11→5
5013 measured reflections	*l* = −21→21

### Refinement

**Table:**

Refinement on *F*^2^	Secondary atom site location: difference Fourier map
Least-squares matrix: full	Hydrogen site location: inferred from neighbouring sites
*R*[*F*^2^ > 2σ(*F*^2^)] = 0.073	H-atom parameters constrained
*wR*(*F*^2^) = 0.220	*w* = 1/[σ^2^(*F*_o_^2^) + (0.118*P*)^2^ + 1.3115*P*] where *P* = (*F*_o_^2^ + 2*F*_c_^2^)/3
*S* = 1.18	(Δ/σ)_max_ < 0.001
4177 reflections	Δρ_max_ = 0.92 e Å^−^^3^
337 parameters	Δρ_min_ = −0.85 e Å^−^^3^
0 restraints	Extinction correction: *SHELXL97* (Sheldrick, 2008), Fc^*^=kFc[1+0.001xFc^2^λ^3^/sin(2θ)]^-1/4^
Primary atom site location: structure-invariant direct methods	Extinction coefficient: 0.027 (5)

### Special details

**Table:**

Geometry. All e.s.d.'s (except the e.s.d. in the dihedral angle between two l.s. planes) are estimated using the full covariance matrix. The cell e.s.d.'s are taken into account individually in the estimation of e.s.d.'s in distances, angles and torsion angles; correlations between e.s.d.'s in cell parameters are only used when they are defined by crystal symmetry. An approximate (isotropic) treatment of cell e.s.d.'s is used for estimating e.s.d.'s involving l.s. planes.
Refinement. Refinement of *F*^2^ against ALL reflections. The weighted *R*-factor *wR* and goodness of fit *S* are based on *F*^2^, conventional *R*-factors *R* are based on *F*, with *F* set to zero for negative *F*^2^. The threshold expression of *F*^2^ > σ(*F*^2^) is used only for calculating *R*-factors(gt) *etc*. and is not relevant to the choice of reflections for refinement. *R*-factors based on *F*^2^ are statistically about twice as large as those based on *F*, and *R*- factors based on ALL data will be even larger.

### Fractional atomic coordinates and isotropic or equivalent isotropic displacement parameters (Å^2^)

**Table:**

	*x*	*y*	*z*	*U*_iso_*/*U*_eq_	
Zn1	0.65848 (11)	0.46816 (9)	0.25386 (4)	0.0402 (4)	
Cl1	0.4662 (3)	0.3566 (2)	0.34307 (9)	0.0479 (5)	
Cl2	0.5032 (3)	0.5538 (2)	0.17408 (10)	0.0491 (5)	
Cl3	0.7041 (4)	0.2815 (3)	0.19234 (12)	0.0686 (7)	
Cl4	0.9008 (3)	0.6651 (3)	0.30484 (12)	0.0639 (6)	
O1	0.2911 (7)	1.1282 (5)	0.4863 (3)	0.0443 (12)	
O2	1.2012 (6)	0.4167 (5)	0.0191 (3)	0.0447 (12)	
N1	0.3863 (7)	0.9990 (6)	0.3799 (3)	0.0362 (13)	
H1	0.3811	1.0809	0.3972	0.043*	
N2	1.1219 (7)	0.2271 (6)	0.1233 (3)	0.0403 (14)	
H2	1.1914	0.3149	0.1088	0.048*	
C7	0.2315 (8)	0.9970 (7)	0.5249 (4)	0.0339 (15)	
C11	0.2044 (9)	0.7392 (8)	0.5306 (4)	0.0383 (16)	
C19	0.8652 (9)	−0.0063 (8)	0.1046 (4)	0.0400 (17)	
C5	0.3231 (8)	0.8696 (7)	0.4211 (4)	0.0334 (15)	
C22	1.0506 (9)	0.3249 (8)	−0.0213 (4)	0.0371 (16)	
C4	0.3342 (9)	0.7386 (7)	0.3914 (4)	0.0367 (16)	
C6	0.2526 (8)	0.8717 (7)	0.4925 (3)	0.0307 (14)	
C20	0.9774 (8)	0.1375 (7)	0.0789 (4)	0.0322 (15)	
C18	0.9075 (10)	−0.0513 (8)	0.1711 (4)	0.0471 (19)	
H18	0.8338	−0.1454	0.1883	0.056*	
C1	0.4544 (10)	1.0034 (9)	0.3153 (4)	0.0453 (18)	
H1A	0.4953	1.0933	0.2903	0.054*	
C13	0.2790 (10)	0.6069 (8)	0.4329 (5)	0.0493 (19)	
H13	0.2863	0.5195	0.4136	0.059*	
C21	0.9408 (8)	0.1852 (8)	0.0091 (4)	0.0348 (15)	
C26	0.7838 (9)	0.0852 (8)	−0.0321 (4)	0.0418 (17)	
C10	0.1336 (10)	0.7295 (9)	0.5993 (4)	0.0486 (18)	
H10	0.1017	0.6412	0.6251	0.058*	
C3	0.4010 (10)	0.7407 (9)	0.3237 (4)	0.0468 (18)	
H3	0.4039	0.6521	0.3035	0.056*	
C8	0.1616 (10)	0.9881 (8)	0.5903 (4)	0.0447 (18)	
H8	0.1463	1.0710	0.6106	0.054*	
C9	0.1130 (11)	0.8546 (10)	0.6269 (4)	0.058 (2)	
H9	0.0645	0.8492	0.6719	0.070*	
C17	1.0585 (11)	0.0437 (10)	0.2115 (4)	0.055 (2)	
H17	1.0911	0.0117	0.2546	0.066*	
C2	0.4640 (10)	0.8740 (9)	0.2854 (4)	0.0488 (19)	
H2A	0.5118	0.8758	0.2405	0.059*	
C14	0.2706 (11)	1.2609 (8)	0.5156 (4)	0.0487 (19)	
H14A	0.1477	1.2350	0.5198	0.073*	
H14B	0.3188	1.3432	0.4834	0.073*	
H14C	0.3318	1.2922	0.5629	0.073*	
C29	1.3189 (10)	0.5596 (8)	−0.0091 (4)	0.0451 (17)	
H29A	1.3556	0.5398	−0.0556	0.068*	
H29B	1.4203	0.6093	0.0243	0.068*	
H29C	1.2591	0.6247	−0.0149	0.068*	
C12	0.2156 (10)	0.6040 (8)	0.5000 (4)	0.0455 (18)	
H12	0.1797	0.5156	0.5260	0.055*	
C16	1.1615 (10)	0.1866 (9)	0.1877 (4)	0.0491 (19)	
H16	1.2586	0.2545	0.2167	0.059*	
C25	0.7424 (10)	0.1275 (9)	−0.1000 (4)	0.0482 (19)	
H25	0.6393	0.0628	−0.1268	0.058*	
C23	1.0090 (10)	0.3623 (9)	−0.0891 (4)	0.0457 (18)	
H23	1.0846	0.4524	−0.1090	0.055*	
C28	0.7095 (10)	−0.1024 (9)	0.0607 (5)	0.052 (2)	
H28	0.6328	−0.1964	0.0770	0.062*	
C27	0.6748 (10)	−0.0574 (9)	−0.0032 (5)	0.050 (2)	
H27	0.5735	−0.1227	−0.0309	0.060*	
C24	0.8520 (10)	0.2638 (10)	−0.1279 (4)	0.051 (2)	
H24	0.8216	0.2906	−0.1729	0.061*	

### Atomic displacement parameters (Å^2^)

**Table:**

	*U*^11^	*U*^22^	*U*^33^	*U*^12^	*U*^13^	*U*^23^
Zn1	0.0497 (6)	0.0382 (5)	0.0349 (5)	0.0200 (4)	0.0069 (4)	0.0055 (3)
Cl1	0.0652 (12)	0.0475 (11)	0.0383 (10)	0.0292 (9)	0.0152 (8)	0.0111 (8)
Cl2	0.0621 (12)	0.0488 (11)	0.0392 (10)	0.0258 (9)	0.0029 (8)	0.0073 (8)
Cl3	0.1108 (19)	0.0549 (13)	0.0580 (13)	0.0491 (13)	0.0347 (12)	0.0140 (10)
Cl4	0.0558 (13)	0.0597 (14)	0.0627 (13)	0.0122 (10)	−0.0040 (10)	0.0037 (11)
O1	0.066 (3)	0.034 (3)	0.044 (3)	0.031 (2)	0.010 (2)	0.002 (2)
O2	0.042 (3)	0.032 (3)	0.047 (3)	0.002 (2)	0.002 (2)	0.008 (2)
N1	0.042 (3)	0.036 (3)	0.035 (3)	0.021 (3)	0.002 (2)	0.004 (2)
N2	0.043 (3)	0.033 (3)	0.038 (3)	0.009 (3)	0.004 (3)	0.006 (3)
C7	0.030 (3)	0.032 (4)	0.042 (4)	0.015 (3)	0.002 (3)	0.005 (3)
C11	0.035 (4)	0.039 (4)	0.041 (4)	0.016 (3)	−0.004 (3)	0.005 (3)
C19	0.040 (4)	0.035 (4)	0.048 (4)	0.017 (3)	0.012 (3)	0.002 (3)
C5	0.034 (4)	0.030 (4)	0.040 (4)	0.018 (3)	−0.002 (3)	−0.002 (3)
C22	0.036 (4)	0.033 (4)	0.040 (4)	0.012 (3)	0.008 (3)	0.003 (3)
C4	0.036 (4)	0.027 (4)	0.046 (4)	0.013 (3)	−0.004 (3)	−0.002 (3)
C6	0.028 (3)	0.027 (3)	0.036 (3)	0.010 (3)	−0.001 (3)	0.007 (3)
C20	0.028 (3)	0.027 (3)	0.039 (4)	0.011 (3)	0.003 (3)	−0.005 (3)
C18	0.046 (4)	0.034 (4)	0.058 (5)	0.013 (4)	0.020 (4)	0.008 (4)
C1	0.054 (5)	0.048 (4)	0.040 (4)	0.027 (4)	0.003 (3)	0.010 (3)
C13	0.049 (4)	0.036 (4)	0.071 (5)	0.026 (4)	0.004 (4)	−0.006 (4)
C21	0.027 (3)	0.034 (4)	0.042 (4)	0.011 (3)	0.005 (3)	−0.002 (3)
C26	0.034 (4)	0.041 (4)	0.050 (4)	0.015 (3)	0.013 (3)	−0.003 (3)
C10	0.056 (5)	0.042 (4)	0.051 (4)	0.024 (4)	0.002 (4)	0.010 (4)
C3	0.055 (5)	0.043 (4)	0.049 (4)	0.028 (4)	−0.003 (3)	−0.010 (4)
C8	0.054 (5)	0.037 (4)	0.049 (4)	0.023 (4)	0.015 (4)	0.007 (3)
C9	0.067 (5)	0.067 (6)	0.044 (4)	0.031 (5)	0.018 (4)	0.000 (4)
C17	0.070 (6)	0.064 (6)	0.038 (4)	0.034 (5)	0.010 (4)	0.016 (4)
C2	0.058 (5)	0.063 (5)	0.036 (4)	0.035 (4)	0.003 (3)	−0.005 (4)
C14	0.076 (5)	0.031 (4)	0.051 (4)	0.034 (4)	0.003 (4)	−0.005 (3)
C29	0.043 (4)	0.033 (4)	0.047 (4)	0.004 (3)	0.007 (3)	0.006 (3)
C12	0.054 (5)	0.037 (4)	0.054 (5)	0.027 (4)	0.003 (4)	0.007 (3)
C16	0.041 (4)	0.056 (5)	0.042 (4)	0.012 (4)	0.000 (3)	0.006 (4)
C25	0.038 (4)	0.050 (5)	0.047 (4)	0.012 (4)	−0.006 (3)	−0.004 (4)
C23	0.046 (4)	0.044 (4)	0.041 (4)	0.013 (4)	0.008 (3)	0.007 (3)
C28	0.041 (4)	0.034 (4)	0.070 (6)	0.005 (3)	0.014 (4)	0.013 (4)
C27	0.034 (4)	0.036 (4)	0.064 (5)	−0.001 (3)	0.007 (3)	−0.006 (4)
C24	0.054 (5)	0.060 (5)	0.037 (4)	0.023 (4)	−0.009 (3)	0.003 (4)

### Geometric parameters (Å, °)

**Table:**

Zn1—Cl4	2.252 (2)	C1—H1A	0.9300
Zn1—Cl3	2.268 (2)	C13—C12	1.359 (11)
Zn1—Cl1	2.305 (2)	C13—H13	0.9300
Zn1—Cl2	2.310 (2)	C21—C26	1.425 (10)
O1—C7	1.380 (8)	C26—C25	1.398 (11)
O1—C14	1.449 (8)	C26—C27	1.426 (11)
O2—C22	1.364 (8)	C10—C9	1.373 (11)
O2—C29	1.442 (8)	C10—H10	0.9300
N1—C1	1.332 (9)	C3—C2	1.392 (11)
N1—C5	1.395 (9)	C3—H3	0.9300
N1—H1	0.8600	C8—C9	1.381 (11)
N2—C16	1.327 (9)	C8—H8	0.9300
N2—C20	1.365 (8)	C9—H9	0.9300
N2—H2	0.8600	C17—C16	1.378 (11)
C7—C8	1.348 (10)	C17—H17	0.9300
C7—C6	1.410 (9)	C2—H2A	0.9300
C11—C6	1.389 (9)	C14—H14A	0.9600
C11—C12	1.443 (10)	C14—H14B	0.9600
C11—C10	1.405 (10)	C14—H14C	0.9600
C19—C18	1.389 (11)	C29—H29A	0.9600
C19—C20	1.417 (10)	C29—H29B	0.9600
C19—C28	1.432 (11)	C29—H29C	0.9600
C5—C4	1.399 (9)	C12—H12	0.9300
C5—C6	1.463 (9)	C16—H16	0.9300
C22—C21	1.421 (9)	C25—C24	1.376 (11)
C22—C23	1.376 (10)	C25—H25	0.9300
C4—C3	1.381 (10)	C23—C24	1.401 (11)
C4—C13	1.414 (10)	C23—H23	0.9300
C20—C21	1.437 (10)	C28—C27	1.326 (12)
C18—C17	1.373 (11)	C28—H28	0.9300
C18—H18	0.9300	C27—H27	0.9300
C1—C2	1.381 (10)	C24—H24	0.9300
			
Cl4—Zn1—Cl3	116.13 (10)	C21—C26—C27	118.7 (7)
Cl4—Zn1—Cl1	109.32 (8)	C9—C10—C11	117.8 (7)
Cl3—Zn1—Cl1	108.22 (8)	C9—C10—H10	121.1
Cl4—Zn1—Cl2	111.04 (8)	C11—C10—H10	121.1
Cl3—Zn1—Cl2	107.14 (8)	C4—C3—C2	120.8 (6)
Cl1—Zn1—Cl2	104.30 (8)	C4—C3—H3	119.6
C7—O1—C14	118.7 (5)	C2—C3—H3	119.6
C22—O2—C29	119.1 (5)	C7—C8—C9	119.3 (7)
C1—N1—C5	123.9 (6)	C7—C8—H8	120.3
C1—N1—H1	118.1	C9—C8—H8	120.3
C5—N1—H1	118.1	C10—C9—C8	122.4 (7)
C16—N2—C20	123.9 (6)	C10—C9—H9	118.8
C16—N2—H2	118.0	C8—C9—H9	118.8
C20—N2—H2	118.0	C18—C17—C16	119.8 (7)
C8—C7—O1	123.1 (6)	C18—C17—H17	120.1
C8—C7—C6	121.3 (6)	C16—C17—H17	120.1
O1—C7—C6	115.6 (6)	C1—C2—C3	118.9 (7)
C6—C11—C12	121.7 (6)	C1—C2—H2A	120.5
C6—C11—C10	120.8 (6)	C3—C2—H2A	120.5
C12—C11—C10	117.4 (7)	O1—C14—H14A	109.5
C18—C19—C20	119.9 (6)	O1—C14—H14B	109.5
C18—C19—C28	121.4 (7)	H14A—C14—H14B	109.5
C20—C19—C28	118.7 (7)	O1—C14—H14C	109.5
C4—C5—N1	116.5 (6)	H14A—C14—H14C	109.5
C4—C5—C6	121.7 (6)	H14B—C14—H14C	109.5
N1—C5—C6	121.7 (5)	O2—C29—H29A	109.5
O2—C22—C21	116.4 (6)	O2—C29—H29B	109.5
O2—C22—C23	122.2 (6)	H29A—C29—H29B	109.5
C21—C22—C23	121.4 (6)	O2—C29—H29C	109.5
C3—C4—C5	120.1 (7)	H29A—C29—H29C	109.5
C3—C4—C13	121.5 (6)	H29B—C29—H29C	109.5
C5—C4—C13	118.4 (6)	C13—C12—C11	119.5 (7)
C11—C6—C7	118.3 (6)	C13—C12—H12	120.3
C11—C6—C5	116.5 (6)	C11—C12—H12	120.3
C7—C6—C5	125.1 (6)	N2—C16—C17	119.8 (7)
N2—C20—C19	116.7 (6)	N2—C16—H16	120.1
N2—C20—C21	122.0 (6)	C17—C16—H16	120.1
C19—C20—C21	121.3 (6)	C24—C25—C26	120.9 (7)
C17—C18—C19	119.7 (7)	C24—C25—H25	119.6
C17—C18—H18	120.2	C26—C25—H25	119.6
C19—C18—H18	120.2	C24—C23—C22	119.6 (7)
N1—C1—C2	119.8 (7)	C24—C23—H23	120.2
N1—C1—H1A	120.1	C22—C23—H23	120.2
C2—C1—H1A	120.1	C27—C28—C19	119.9 (7)
C12—C13—C4	122.1 (6)	C27—C28—H28	120.0
C12—C13—H13	118.9	C19—C28—H28	120.0
C4—C13—H13	118.9	C28—C27—C26	123.8 (7)
C22—C21—C20	124.4 (6)	C28—C27—H27	118.1
C22—C21—C26	118.0 (6)	C26—C27—H27	118.1
C20—C21—C26	117.6 (6)	C25—C24—C23	120.6 (7)
C25—C26—C21	119.5 (7)	C25—C24—H24	119.7
C25—C26—C27	121.8 (7)	C23—C24—H24	119.7

### Hydrogen-bond geometry (Å, °)

**Table:**

*D*—H···*A*	*D*—H	H···*A*	*D*···*A*	*D*—H···*A*
N1—H1···O1	0.86	1.95	2.612 (7)	133
N1—H1···Cl1^i^	0.86	2.68	3.319 (6)	132
N2—H2···O2	0.86	1.93	2.598 (7)	134
N2—H2···Cl2^ii^	0.86	2.84	3.472 (6)	132

Symmetry codes: (i) *x*, *y*+1, *z*; (ii) *x*+1, *y*, *z*.
